# Floral organ-specific proteome profiling of the floral ornamental orchid (*Cymbidium goeringii*) reveals candidate proteins related to floral organ development

**DOI:** 10.1186/s40529-021-00330-9

**Published:** 2021-12-18

**Authors:** Yue Chen, Zihan Xu, Qi Shen, Chongbo Sun

**Affiliations:** 1 Institute of Horticulture, Zhejiang Academy of Agriculture Science, Hangzhou, Zhejiang China; 2 Plant Protection and Microbiology, Zhejiang Academy of Agricultural Science, Hangzhou, Zhejiang China; 3 College of Landscape and Architecture, Nanjing Forestry University, Nanjing, Jiangsu China

**Keywords:** *Cymbidium*, Floral organ, Proteomic analysis, Tandem Mass Tag, Transcription factor

## Abstract

**Background:**

*Cymbidium goeringii*, belonging to the Orchidaceae family, is an important ornamental plant with striking petals and lips. Extremely diversified floral patterns and morphologies make *C. goeringii* good research material to examine floral development of orchids. However, no floral organ-specific protein has been identified yet. To screen floral development associated proteins, four proteomes from petal (PE), lip (LI), gynostemium (GY), and sepal (SE) were analyzed using Tandem Mass Tag-based proteomic analysis.

**Results:**

A total of 6626 unique peptides encoding 2331 proteins were identified in our study. Proteins in several primary metabolic pathways, including amino acid metabolism, energy metabolism, and lipid metabolism, were identified as differentially expressed proteins. Interestingly, most of the energy metabolism-related proteins highly expressed in SE, indicating that SE is an important photosynthetic organ of *C. goeringii* flower. Furthermore, a number of phytohormone-related proteins and transcription factors (TFs) were identified in *C. goeringii* flowers. Expression analysis showed that 1-aminocyclopropane-1-carboxylate oxidase highly expressed in GY, IAA-amino acid hydrolase ILR1-like 4 and gibberellin receptor 1 C greatly expressed in LI, and auxin-binding protein ABP20 significantly expressed in SE, suggesting a significant role of hormones in the regulation of flower morphogenesis and development. For TFs, GY-highly expressed bHLH13, PE-highly expressed WRKY33, and GY-highly expressed VIP1, were identified.

**Conclusions:**

Mining of floral organ differential expressed enzymes and TFs helps us to excavate candidate proteins related to floral organ development and to accelerate the breeding of *Cymbidium* plants.

**Supplementary Information:**

The online version contains supplementary material available at 10.1186/s40529-021-00330-9.

## Background

Orchidaceae family, the largest group of flowering plants on our planet, contains a number of horticulturally important and favored ornamental plants (de la Torre Llorente 2018). The genus *Cymbidium*, consisting of *C. goeringii*, *C. sinense*, *C. faberi* Rolfe, *C. tortisepalum*, and *C. kanran*, holds a large share of the flower market in China and other Southeast Asian countries (de la Torre Llorente 2018; Wang et al. 2020). *C. goeringii*, which is called the spring orchid, blooms from later January to early March; consequently, it is a widely required Spring Festival gift (Yang et al. 2017). Extremely diversified floral morphological patterns and colors make *C. goeringii* substantial commercial value (Yang et al. 2013).

With increased demand, many fascinating new orchid cultivars with altered floral characteristics have been developed by cross-breeding or mutation-breeding annually (Xiang et al. 2018). Many studies on *C. goeringii* floral trait-related genes, have been performed (Huang et al. 2012). For example, the *FLOWERING LOCUS T* (FT) orthologs from *C. goeringii* have been functional identified as a regulators of the vegetative to reproductive transition (Xiang et al. 2012). The low-temperature-induced transcriptomes of *C. goeringii* revealed that *CgSVP* gene plays an essential role in the regulation of flowering by interacting with two important flowering regulators, CgAP1 and CgSOC1 (Yang et al. 2019). Screening of a *C. goeringii* cDNA library identified a number of B and E class MADS-box genes, including *AP*-, *AP2*-, *SEP*-, *DEF*-, *GLO*- and *AGL6*-like genes, involving in the determination of perianth formation (Xiang et al. 2018). A recent RNA sequencing analysis of *C. goeringii* identified several floral scent biosynthesis-related genes (Ramya et al. 2019). However, no proteome of *C. goeringii* has been reported to date. Many flowering and floral development-related genes display floral organ-specific expression patterns (Suzuki et al. 2017). For example, a zinc finger protein EPF1, which is involved in the expression of the 5-enolpyruvylshikimate-3-phosphate synthase-encoding gene, is specifically expressed in *petunia* petals (Takatsuji et al. 1994). Several floral organ-specific promoters, such as *Arabidopsis TCP3* and tobacco *AP1-like*, function in the regulation of floral transition, initiation and development (Katsutomo et al. 2016; Zhang et al. 2014). Furthermore, some floral organ-related hormones displayed organ-specific accumulation (Liu et al. 2013). In *Arabidopsis*, stamen development is controlled by organ-specific over-expression of ethylene synthesis gene *CsACO2* (Duan et al. 2008). Thus, screening of floral organ-specific genes and proteins may help us to identify the regulation factors that are involved in floral development.

Recently, a gel-free tandem mass spectrometry (MS/MS)-based proteomics method with isobaric labeling reagents has been developed for accurate quantification of proteins (Smolikova et al. 2020). Liquid chromatograph (LC)-MS/MS based comparative proteomics of floral nectars revealed the differential expressed proteins involved in floral defense of various plant species, such as *Nicotiana* spp., *Petunia hybrida* and *Datura stramonium* (Silva et al. 2020). Using the gel-free MS/MS-based proteomics method, a large number of proteins can be obtained and more valuable genetic information can be mined (Hao et al. 2017). Compared with the traditional 2-D method, the quantitative analysis of protein accumulation level is also more accurate, which is helpful to screen differentially expressed proteins among floral organs. In our study, floral organ-specific proteome profiling of metabolic pathways and transcription factors may help to accelerate the breeding of *Cymbidium* plants.

## Results

### Overview of the Tandem Mass Tag (TMT) LC-MS/MS data

**Fig. 1:**
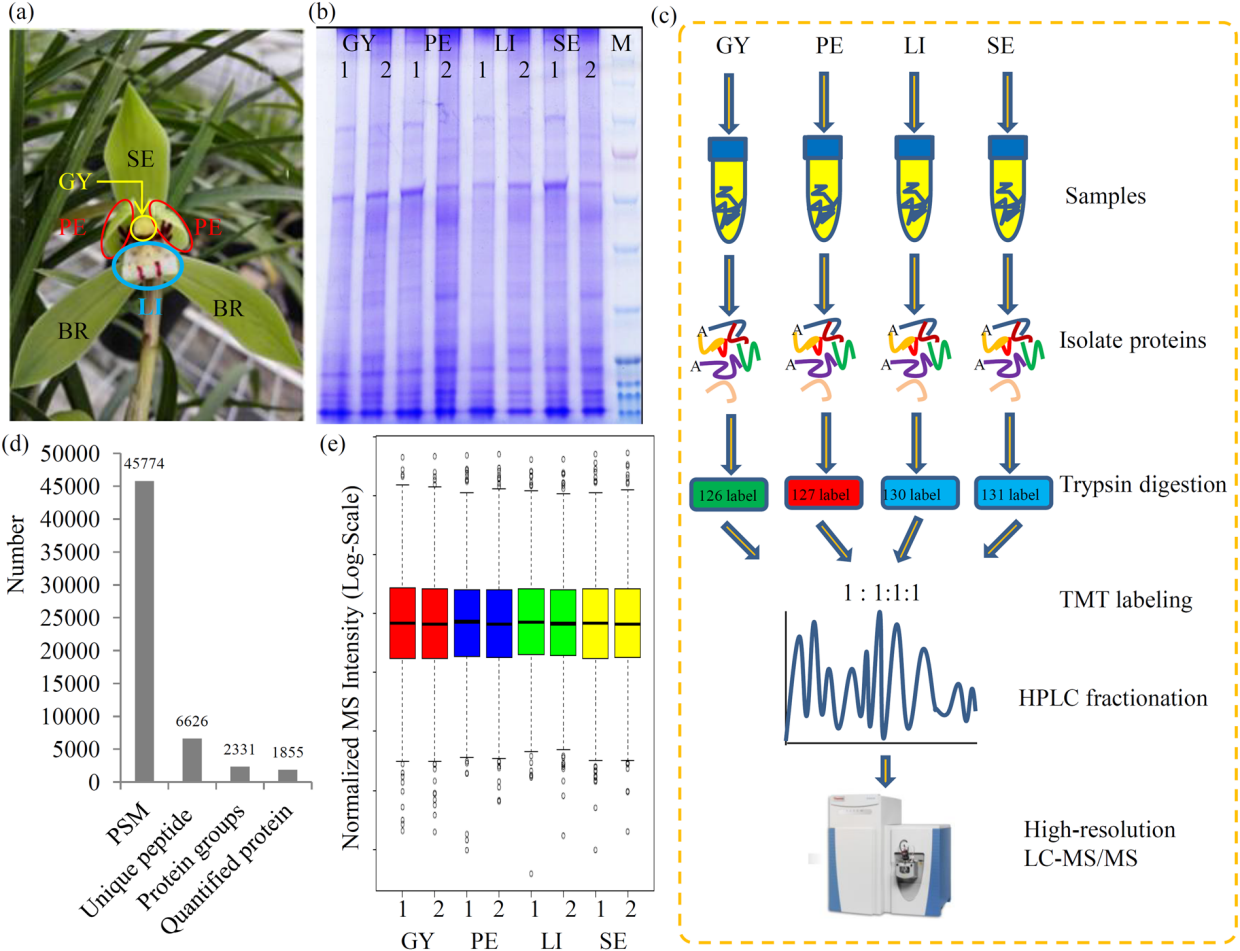
Overview of the proteomic data. **a** A picture of typical *Cymbidium goeringii* flower. Samples from different floral organs, including gynostemium (GY): petal (PE), lip (LI), and sepal (SE) are showed in the picture. **b** The SDS-PAGE Gel electrophoretic graph for the samples from four different organ groups, including GY, PE, LI, and SE. M: protein marker. **c** Experimental strategy for the quantitative analysis of proteomes from four different floral organs. **d** Statistic results of protein identification. **e** Normalized MS intensity

A picture showed the detail sites of the four floral organs, Sepal (SE), petal (PE), lip (LI), and gynostemium (GY) (Fig. 1a). The protein samples were extracted from the four floral organs (Fig. 1b). The basic chart of our experiment process is shown in Fig. 1c. In total, 45,774 peptide-spectral matches, 6626 unique peptides, 2331 protein groups, and 1,855 quantified proteins, were obtained (Fig. 1d). A boxplot of normalized densities are shown in Fig. 1e. The basic information for all the identified proteins, including protein IDs, protein annotations, peptide numbers, unique peptide numbers, sequence coverages, molecular weights, and sequence lengths, are listed in Additional file 1: Table S1.

### Identification of the differential expressed proteins (DEPs) between different sample groups

**Fig. 2:**
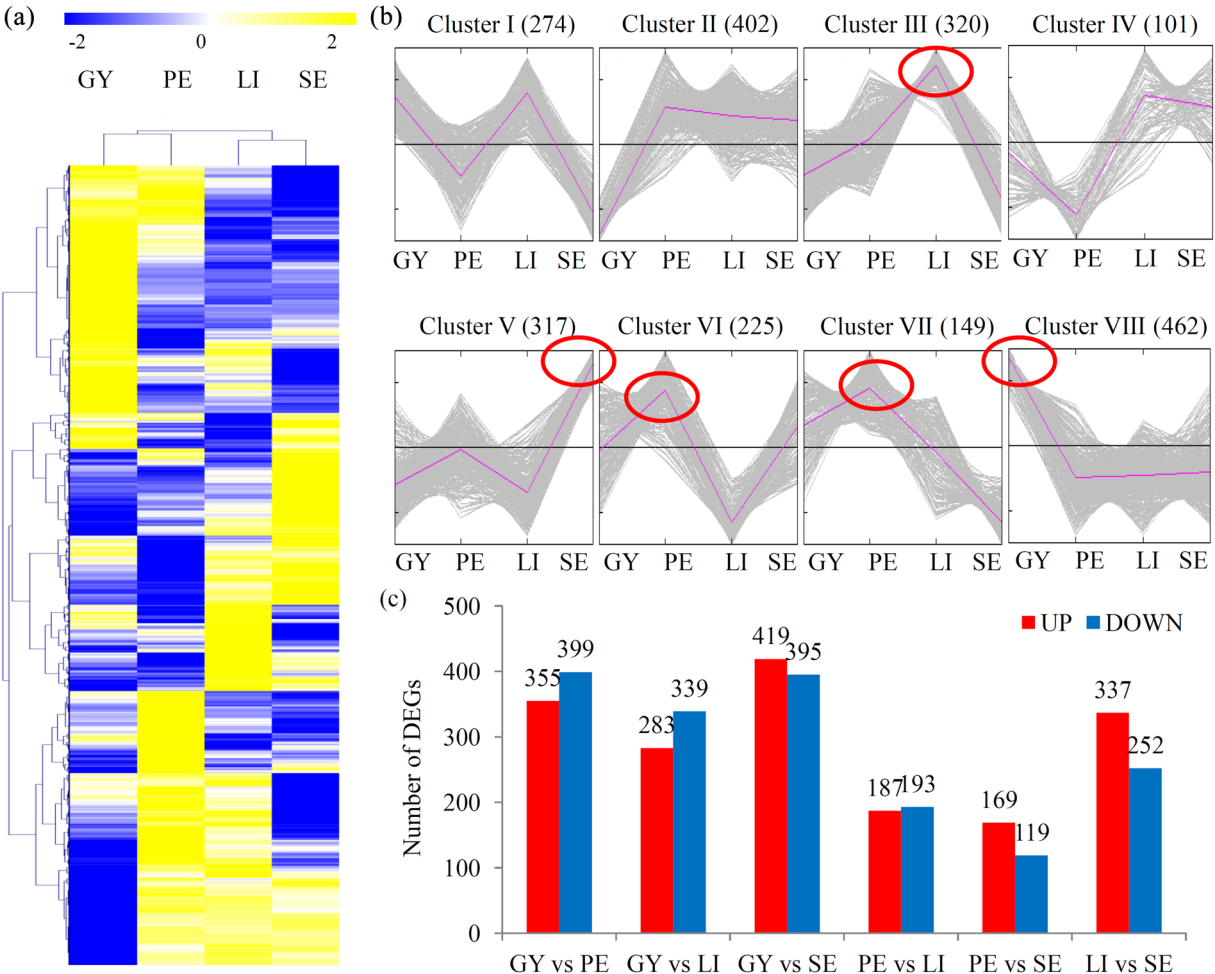
Analysis of differentially expressed proteins (DEPs) among the four floral organs. **a** A heatmap of the abundance of proteins in the four floral organs (*N* = 2). The heatmap scale ranges from − 2 to + 2 on a *log*_*2*_ scale. **b**–**l** MeV cluster analysis showed the numbers of floral organ-specific expressed proteins. The red oval indicated the number of organ specific expressed proteins. **j** The number of DEPs in different comparisons, including the GY vs. PE, GY vs. LI, GY vs. SE, PE vs. LI, PE vs. SE, LI vs. SE comparisons

Proteomic profiles of the floral organs, including GY, PE, LI and SE, is shown in Fig. 2a. All identified proteins were grouped into different clusters. In detail, the GY highly expressed proteins were grouped into Cluster VIII (462 proteins), the PE significantly expressed proteins were grouped into Clusters VI (225 proteins) and VII (149 proteins), the LI greatly expressed proteins were grouped into Cluster III (320 proteins), and the SE highly expressed proteins were classed into Cluster V (317 proteins) (Fig. 2b).

The DEPs among different sample groups were analyzed and counted. In detail, 355 up- and 399 down-regulated proteins in the GY vs. PE comparison; 283 up- and 339 down-regulated proteins in the GY vs. LI comparison, 419 up- and 395 down-regulated proteins in the GY vs. SE comparison, 187 up- and 193 down-regulated proteins in the PE vs. LI comparison, 169 up- and 119 down-regulated proteins in the PE vs. SE comparison, and 337 up- and 252 down-regulated proteins in the LI vs. SE comparison, were identified (Fig. 2c). On the basis of the numbers of DEPs, there were great differences between GY and other three sample groups (PE, LI and SE) and limited differences between PE and two other sample groups (LI and SE).

### Kyoto Encyclopedia of Genes and Genomes (KEGG) enrichment analysis of the DEPs

**Fig. 3:**
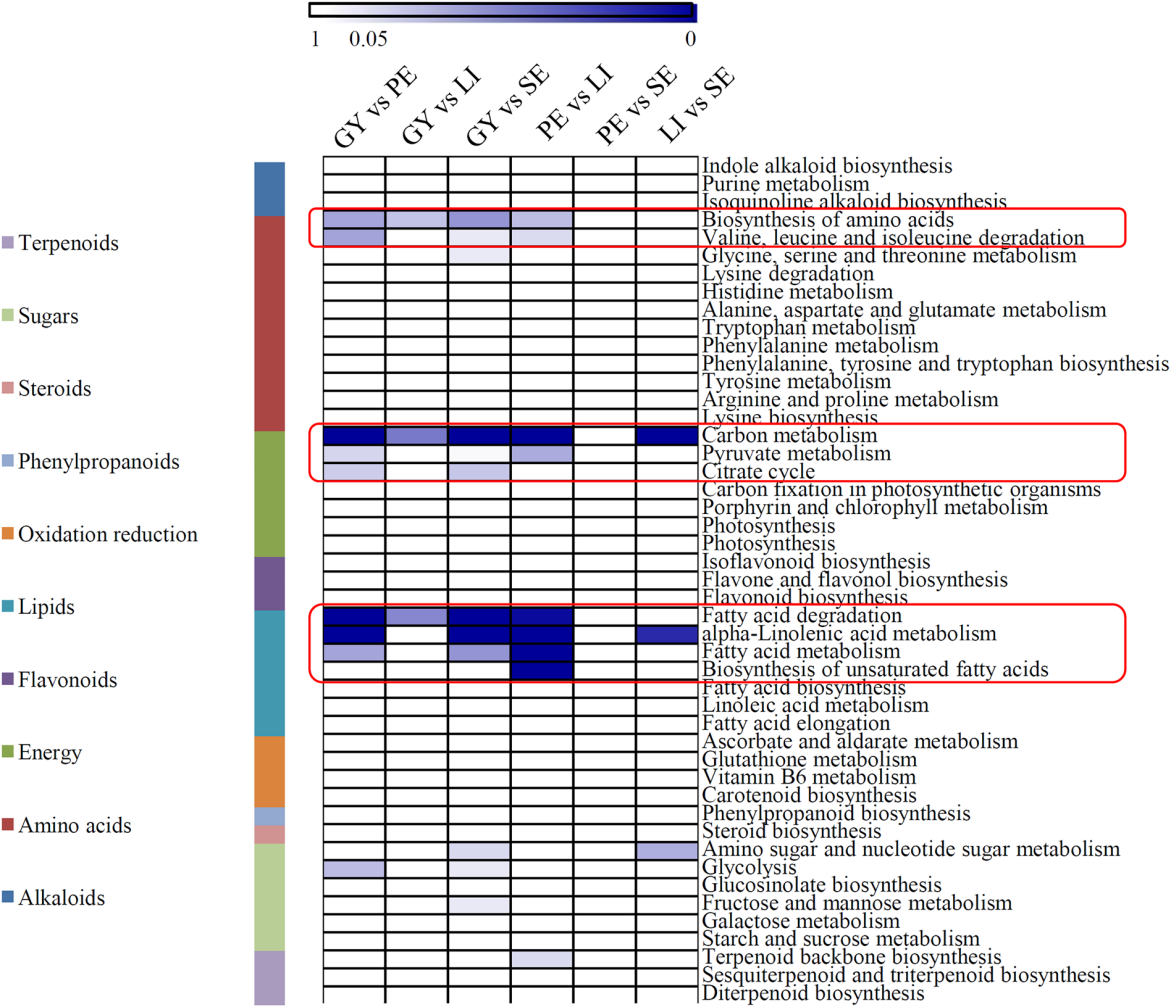
KEGG enrichment analysis of all the DEPs in different comparisons. The significant *P* values of each KEGG term in the six comparisons were shown by heatmap. The heatmap scale ranges from 0 to 1. The red boxes indicated the significant differentially KEGG terms

Most of the DEPs were assigned to 47 metabolic KEGG pathways belonging to 10 major categories. Interestingly, the DEPs in most comparisons were significantly enriched in the primary metabolism-related KEGG categories, including the amino acid-, energy-, and lipid-related categories. In the amino acid category, most of the DEPs were significantly enriched in the ‘biosynthesis of amino acids’ and ‘valine, leucine and isoleucine degradation’ KEGG terms; in the energy category, most of the DEPs were significantly enriched in the ‘carbon metabolism’, ‘pyruvate metabolism’ and ‘citrate cycle’ KEGG terms; and in the lipid category, most of the DEPs were significantly enriched in the ‘fatty acid degradation’, ‘α-linolenic acid metabolism’, ‘fatty acid metabolism’, and ‘biosynthesis of unsaturated fatty acids’ KEGG terms (Fig. 3). Interestingly, no significant KEGG term was identified in the FB vs. SE comparison.

### Differential expression of amino acid metabolism-related proteins

In our study, 17 amino acid metabolism-related enzymes, including aspartate aminotransferase (AST), arogenate dehydratase 2 (ADT2), glycine cleavage system H protein (GCSH), cysteine synthase (CS), anthranilate synthase (ASA2), asparagine synthetase (ASNS), 3-isopropylmalate dehydrogenase 3 (IMD3), serine hydroxymethyltransferase 1 (SHMT1), isovaleryl-CoA dehydrogenase 1 (IVD1), *S*-adenosylmethionine synthase (SAM1), chorismate synthase (ARO2), glutamate–glyoxylate aminotransferase 2 (GGAT2), threonine dehydratase (ILV1), 3-isopropylmalate dehydratase 1 (LEU1), d-3-phosphoglycerate dehydrogenase (PHGDH), dihydrolipoyl dehydrogenase (DLD), and amino-methyltransferase (AMT) were identified in orchid (Additional file 2: Table S2).

**Fig. 4:**
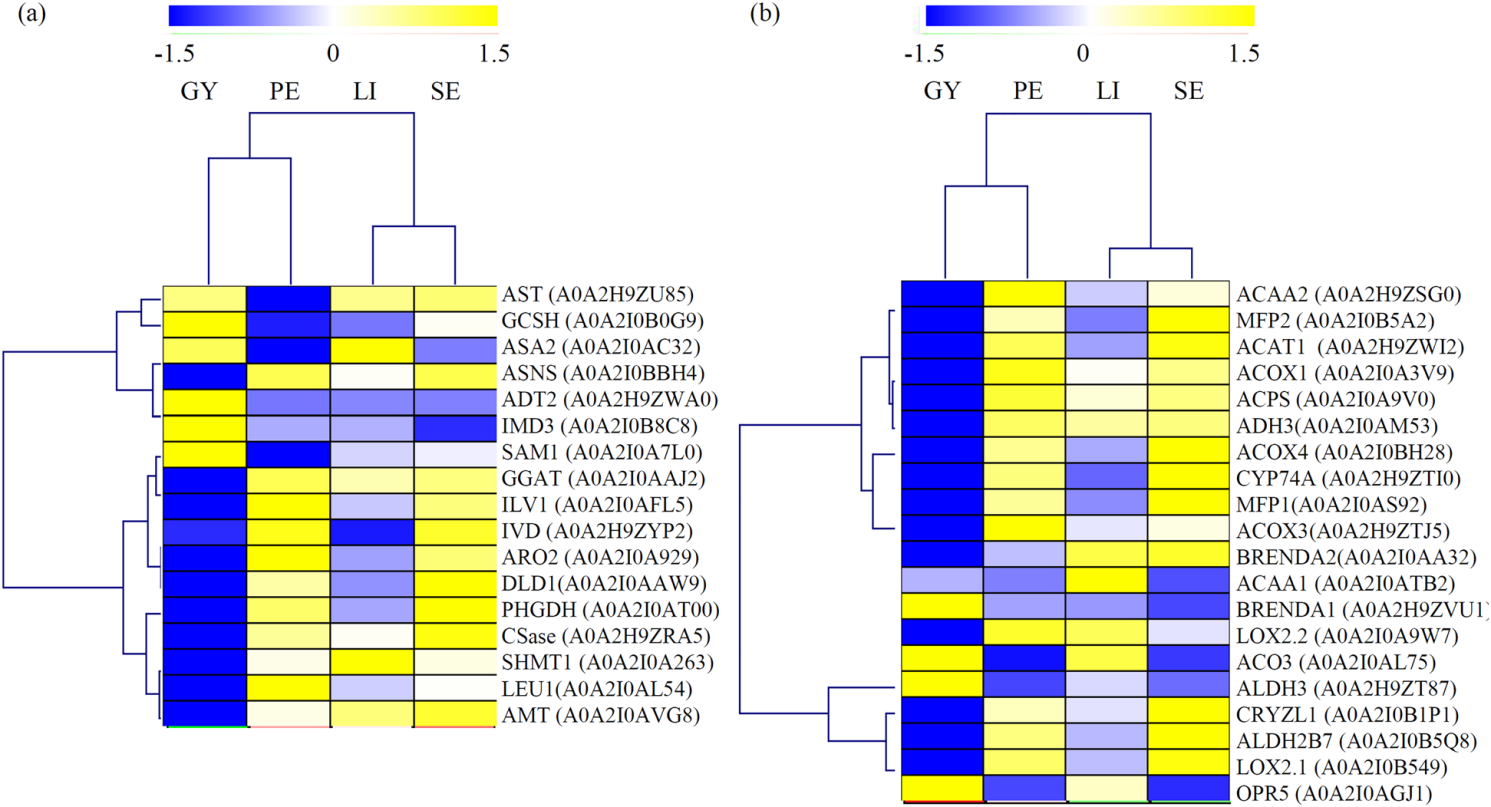
Identification of amino acid metabolism- and lipid metabolism-related proteins. **a** Differential expression of amino acid metabolism-related proteins among four different floral organs. **b** Differential expression of lipid metabolism-related proteins among four different floral organs. The heatmap scale ranges from − 1.5 to 1.5

Among the amino acid metabolism-related proteins, ASA2, ASNS, ADT2 and IMD3 highly expressed in the GY; SAM1, GGAT, ILV1, IVD, and ARO2 greatly expressed in the PE and LI; PHGDH highly expressed in the LI; and CSase, SHMT1, LEU1 and AMT lowly expressed in the GY (Fig. 4a).

### Differential expression of lipid metabolism-related proteins

Our study identified 19 fatty acid metabolism-related proteins, including 3-ketoacyl-CoA thiolase 2 (ACAA2), acyl-coenzyme A oxidase 3 (ACOX3), Acyl-[acyl-carrier-protein] desaturase 1 (BRENDA1), acetyl-CoA acetyltransferase (ACAT1), acyl-coenzyme A oxidase 3 (ACOX1), 3-oxoacyl-[acyl-carrier-protein] synthase (ACPS), Acyl-[acyl-carrier-protein] desaturase 2 (BRENDA2), multifunctional protein 1 (MFP1), 3-ketoacyl-CoA thiolase 1 (ACAA1), multifunctional protein 2 (MFP2), acyl-coenzyme A oxidase 4 (ACOX4), aldehyde dehydrogenase 3 (ADH 3), aldehyde dehydrogenase family 2 member B7 (ALDH2B7), alcohol dehydrogenase 3 (ADH3), 1uinone oxidoreductase-like protein 1 (CRYZL1), lipoxygenase 2.1 (LOX2.1), allene oxide synthase (CYP74A), lipoxygenase 2.2 (LOX2.2), Allene oxide cyclase 3 (AOC3), and 12-oxophytodienoate reductase 5 (OPR5), were identified in orchid (Additional file 3: Table S3).

Among the lipid metabolism-related proteins, ALDH2B7, LOX2.1 and OPR5 predominantly expressed in the GY sample; ALDH3 highly expressed in the PE sample; and most of these proteins, such as ACAA2, MEP2, ACAT1, ACOX1, ACPS, ADH3, ACOX4, CYP74A, MFP1, ACOX3, BRENDA2, ACAA1, BRENDA1, LOX2.2, and LOX2.2, greatly expressed in the LI and SE samples (Fig. 4b).

### Differential expression of energy metabolism-related proteins

**Fig. 5:**
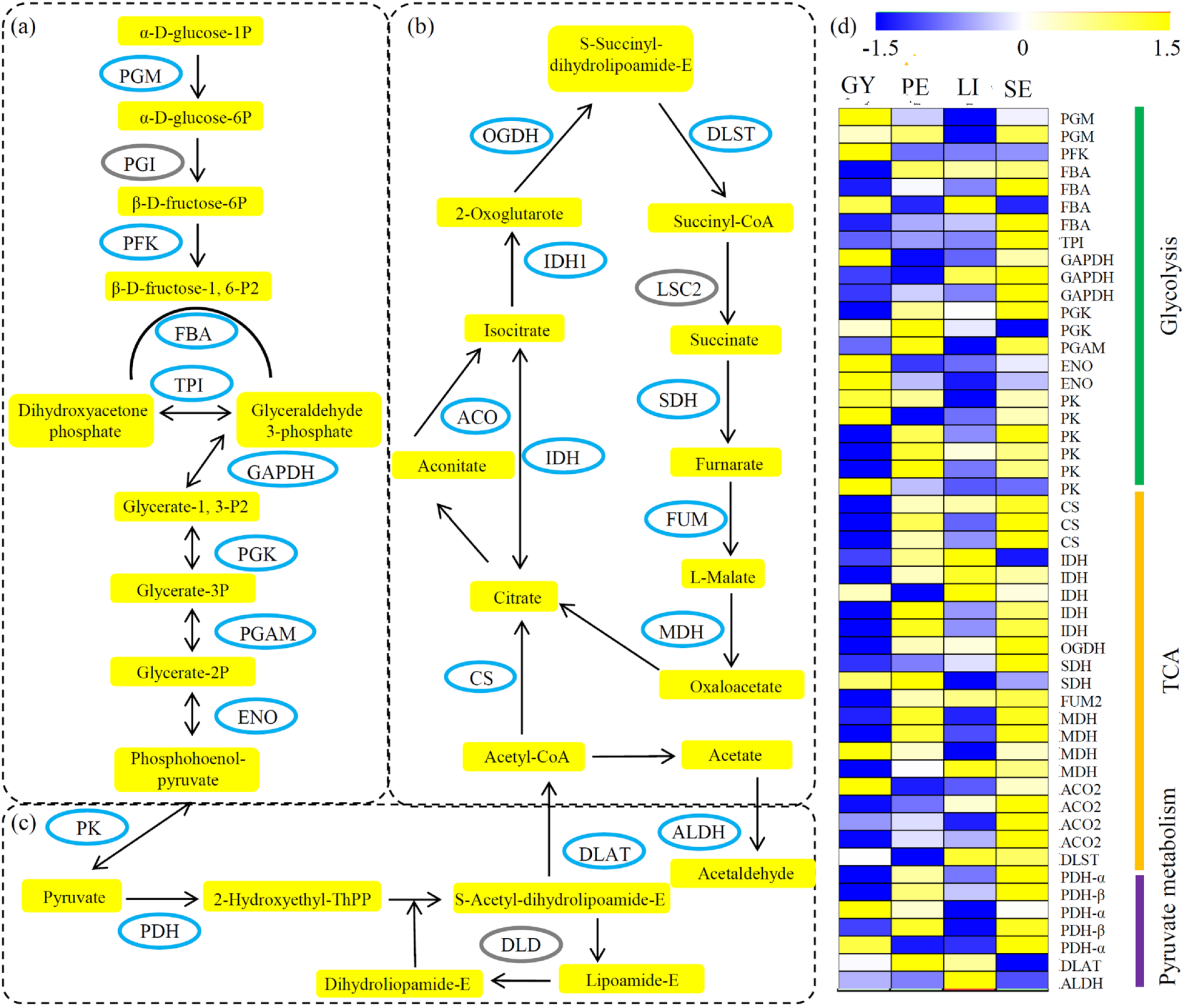
Identification of energy metabolism-related proteins. **a** Identification of glycolysis-related proteins. **b** Differential expression of TCA cycle-related proteins among four different floral organs. **c** Differential expression of pyruvate metabolic pathway-related proteins among four different floral organs. **d** Differential expression of energy metabolism-related proteins among four different floral organs. The heatmap scale ranges from − 1.5 to 1.5

In the glycolysis pathway, a number of proteins, including two phosphoglucomutases (PGMs), one 6-phosphofructokinase (PFK), four fructose-bisphosphate aldolases (FBAs), one triosephosphate isomerase (TPI), three glyceraldehyde-3-phosphate dehydrogenases (GAPDHs), two phosphoglycerate kinases (PGKs), one phosphoglycerate mutase (PGAM), two enolases (ENOs), and six pyruvate kinases (PKs), were identified in orchid (Fig. 5a and Additional file 4: Table S4); for the TCA cycle, three citrate synthases (CSs), five isocitrate dehydrogenases (IDHs), one 2-oxoglutarate dehydrogenase (OGDH), two succinate dehydrogenases (SDHs), one fumarate hydratase 2 (FUM2), four malate dehydrogenases (MDHs), four aconitate hydratase 2 (ACO2) proteins, and one dihydrolipoyllysine-residue succinyltransferase (DLST), were identified in orchid (Fig. 5b and Additional file 4: Table S4); for the pyruvate metabolic pathway, three pyruvate dehydrogenase (PDH)-α subunits, two PDH-β subunits, one dihydrolipoamide acetyltransferase (DLAT), and one aldehyde dehydrogenase (ALDH), were identified in orchid (Fig. 5c and Additional file 4: Table S4).

The expression patterns of the energy metabolism-related proteins are shown in Fig. 5d. Interestingly, most of these proteins displayed high expression levels in the SE sample.

### Differential expression of hormone-related proteins

Phytohormones play important roles in floral organ development (Stewart et al. 2016). In our study, seven auxin-related proteins, including one auxin-binding protein (ABP20), four indole-3-acetic acid (IAA)-amino acid hydrolases (ILLs), one auxin transport protein (BIG), one auxin efflux carrier (PIN3), were identified (Table 1). In the GA signal pathway, one gibberellin-regulated protein 3 and one gibberellin receptor GID1C, were identified. Furthermore, one ethylene signaling pathway-related protein (1-aminocyclopropane-1-carboxylate oxidase 1, ACO1) and one ABA signaling pathway-related protein (abscisic acid receptor, PYL8) were also identified. Expression analysis showed that ACO1 highly expressed in the GY sample, ILL4 and GID1C greatly expressed in the LI sample, and ABP20 significantly expressed in the SE sample.

**Table 1:** Differential expression analysis of hormone-related proteins in differential floral organs of *C. goeringii*

Protein ID	Protein name	GY	PE	LI	SE
A0A2I0A331	ABP20	0.79 ± 0.33	0.96 ± 0.49	0.76 ± 0.26	1.49 ± 0.10
A0A2I0A357	ILL6	0.94 ± 0.09	1.03 ± 0.06	0.82 ± 0.01	1.21 ± 0.03
A0A2I0A842	ILL3	0.64 ± 0.01	1.35 ± 0.05	1.32 ± 0.07	0.70 ± 0.03
A0A2I0AQ01	BIG	1.01 ± 0.09	1.03 ± 0.04	0.88 ± 0.00	1.08 ± 0.05
A0A2I0AS08	ILL3	0.91 ± 0.06	0.97 ± 0.07	1.35 ± 0.02	0.78 ± 0.01
A0A2I0B089	ILL1	0.98 ± 0.03	0.96 ± 0.05	1.15 ± 0.03	0.92 ± 0.05
A0A2I0B9W0	PIN3	0.40 ± 0.09	0.62 ± 0.08	0.50 ± 0.02	0.48 ± 0.01
A0A2I0AT63	GASA3	1.21 ± 0.06	1.10 ± 0.05	1.37 ± 0.00	0.32 ± 0.01
A0A2I0AZC5	GID1C	0.81 ± 0.01	1.17 ± 0.05	1.31 ± 0.04	0.70 ± 0.00
A0A2I0ATV2	PYL8	0.95 ± 0.12	1.14 ± 0.09	1.08 ± 0.08	0.83 ± 0.06
A0A2I0B714	ACO1	1.18 ± 0.10	0.21 ± 0.22	0.38 ± 0.04	0.23 ± 0.18

SE: Sepal; PE: petal; LI: lip; GY: gynostemium

### Differential expression of cell division and pigment production

In total, seven cell division-related proteins, including five Cell Division Cycle family proteins, and two Filamenting Temperature-Sensitive mutant Z (FTS-Z) proteins, were identified (Additional file 5: Table S5). Among these cell division-related proteins, no organ-specific expressed proteins were identified. Furthermore, five pigment production-related proteins, including two Isoflavone reductase like (IRL) proteins, and three CYP family proteins, were detected (Additional file 5: Table S5). Expression analysis showed that two IRL proteins highly expressed in the GY.

### Differential expression of transcription factors (TFs)

In total, 15 TFs, including two bHLHs, two CAMTAs, two HBPs, two LWDs, two WRKYs, one AGL9, one BIM2, one MADS2, one PUR1, and one VIP1, were identified (Table 2). Most of these TFs constitutively expressed in the four floral organs. Expression analysis showed that bHLH13 and VIP1 highly expressed in the GY sample and WRKY33 greatly expressed in the PE sample.

**Table 2:** Differential expression analysis of transcription factors in different floral organs of *C. goeringii*

Protein IDs	Protein name	GY	PE	LI	SE
A0A2I0B2N2	AGL9	0.68 ± 0.03	1.12 ± 0.00	1.13 ± 0.02	1.07 ± 0.01
A0A2I0A921	bHLH13	1.44 ± 0.22	0.92 ± 0.15	0.88 ± 0.04	0.76 ± 0.04
A0A2I0BCG8	bHLH66	1.19 ± 0.00	0.90 ± 0.01	1.19 ± 0.04	0.72 ± 0.04
A0A2I0AWA6	BIM2	0.27 ± 0.18	0.61 ± 0.08	0.59 ± 0.14	0.53 ± 0.06
A0A2I0AGP2	CAMTA2	0.58 ± 0.02	0.44 ± 0.06	0.61 ± 0.07	0.37 ± 0.03
A0A2I0A0M6	CAMTA3	0.95 ± 0.03	1.00 ± 0.03	0.96 ± 0.03	1.09 ± 0.09
A0A2I0B539	HBP1α	1.11 ± 0.12	0.98 ± 0.04	1.00 ± 0.04	0.91 ± 0.04
A0A2I0BEK2	HBP1β	1.04 ± 0.19	1.00 ± 0.22	1.21 ± 0.16	0.74 ± 0.13
A0A2I0AV72	LWD1	1.05 ± 0.08	1.02 ± 0.08	0.94 ± 0.06	0.99 ± 0.06
A0A2I0AZ75	LWD2	0.89 ± 0.17	1.16 ± 0.15	1.08 ± 0.14	0.87 ± 0.12
A0A2I0BG95	MADS2	0.59 ± 0.02	1.12 ± 0.15	1.07 ± 0.01	1.22 ± 0.05
A0A2H9ZTZ8	MSI1	0.69 ± 0.07	0.42 ± 0.09	0.48 ± 0.08	0.42 ± 0.09
A0A2I0A8F1	PUR1	0.89 ± 0.05	1.00 ± 0.01	0.84 ± 0.01	1.26 ± 0.03
A0A2I0B155	VIP1	2.12 ± 0.25	0.50 ± 0.14	0.67 ± 0.09	0.71 ± 0.02
A0A2I0APB9	WRKY3	0.80 ± 0.15	1.13 ± 0.06	1.03 ± 0.01	1.04 ± 0.08
A0A2I0BH59	WRKY33	0.24 ± 0.03	1.01 ± 0.43	0.34 ± 0.08	0.42 ± 0.05

SE: Sepal; PE: petal; LI: lip; GY: gynostemium

## Discussion

Owing to its multiple flowering patterns, *C. goeringii* has a high economic value and is widely favored in East Asia (Chung et al. 2011; Hyun et al. 2012). However, limited genetic information on *C. goeringii* is available and the molecular mechanism responsible for floral patterning is also largely unknown. In the present study, we have analyzed the spatial protein expression pattern in flowers by comparing the protein profiles of four floral organs.

Comparative proteomic analyses have been previously applied to identify the DEPs in *Cymbidium* plants (Chen et al. 2018a; Li et al. 2014). Using traditional 2-D technology, a large number of protein spots were detected in *C. ensifolium*, but only 30 differentially expressed spots were excised and analyzed using MALDI-TOF/TOF (Li et al. 2014). Another 2-D analysis identified 103 DEPs and 104 DEPs responsive to drought in *C. sinense* and *C. tracyanum*, respectively (Li et al. 2018). In the present study, we have identified 2331 proteins, which is more than the previously published works. As expected, most of the quantified proteins showed floral organ-specific expression pattern (Fig. 2a). The comprehensive information will help us to investigate novel proteins that are potentially associated with the floral development of *C. goeringii*.

Although the physiological and molecular characteristics of flowers are well-recognized in the model plants, the primary metabolism during the floral development process of orchids is largely unknown (Muller et al. 2010). Most DEPs were enriched in several primary metabolic pathways, such as amino acid metabolism, energy metabolism, and lipid metabolism pathways (Fig. 3), indicating that significant differences in primary metabolism among four different floral organs of *C. goeringii*. In flowering plants, carbohydrates provide energy and precursors for the secondary metabolism in floral organs(Borghi and Fernie 2017) and serve as nutritional rewards for pollinators (Roy et al. 2017). In medicinal *Chrysanthemum*, soluble sugar and amino acid contents were significantly induced during the floral development process (Ma et al. 2016). The cycles of carbohydrate hydrolysis are involved in pollen development, pollen tube growth, and pollination (Pacini et al. 2006). In our study, a number of carbohydrate hydrolysis-related enzymes and their floral organ-specific expression patterns were revealed in *C. goeringii* (Fig. 4). In flowers, photosynthesis mostly occurs in sepals and young petals, and carbon resources are then transferred to the other floral organs (Muller et al. 2010). *C. goeringii* flowers have three large and green sepals (Fig. 1a). Interestingly, most of the energy metabolism-related proteins highly expressed in the SE, indicating that it is an important photosynthetic organ of *C. goeringii* flowers.

In flowering plants, various phytohormones, such as gibberellins (GAs), jasmonates (JAs), auxins, brassinosteroids (BRs), and cytokinins (CKs), play significant roles in the regulation of flower morphogenesis and development (Song et al. 2013). Auxin’s capacity to regulate aspects of growth and development has been deeply characterized in orchids (Novak et al. 2014). For example, auxin plays an essential role in pollination-induced ovary growth and inflorescence initiation in *Dendrobium* orchids (Ketsa et al. 2006). ABP20, an important auxin receptor protein, controls the first event in the auxin action process (Lazarus et al. 1991). In our study, ABP20 highly expressed in the SE sample, suggesting active auxin signal transduction during SE growth. To regulate the indole-3-acetic acid (IAA) levels, IAA-amino acid hydrolase and IAA-amido synthetase function in the permanent inactivation and temporary storage of auxin (LeClere et al. 2002). In *C. goeringii* flowers, three out of four ILLs are ubiquitous in all the floral organs, suggesting an essential role of auxin homeostasis in floral patterning and development (Yamaguchi et al. 2017). GID1 is a soluble GA receptor widely identified in various plant species (Nakajima et al. 2006; Ueguchi-Tanaka et al. 2005). In our study, differential expression analysis showed that the gibberellin receptor GID1C of *C. goeringii* was a LI-specifically expressed protein, suggesting a potential role of GID1C in the growth and development of flower lip. ACO catalyzes the conversion of 1-aminocyclopropane-1-carboxylate (ACC) to ethylene (Nadeau et al. 1993). In *petunia* flowers, the *ACO* gene showed a pistil-specific and ethylene-regulated expression pattern (Sanchez and Mariani 2002). The ACO1 of *C. goeringii* predominantly expressed in the GY organ, indicating a greater level of ethylene in GY than other floral organs. Ethylene plays an important role in the regulation of floral and organ abscission (Kucko et al. 2019). High ethylene accumulation in GY might promote the senescence of floral organs of *C. goeringii*.

Recently, a number of flowering-related TFs, including the MYB, bHLH and C2H2 families, have been identified in model plants (Chen et al. 2018b; Zhou et al. 2019). In *Arabidopsis*, CIB1, a typical bHLH TF, is involved in the regulation of floral induction (Wang et al. 2018) and WRKY75 is a positive regulator of flowering initiation (Zhang et al. 2018). CpWRKY71, a WRKY TF of wintersweet, promotes flowering (Huang et al. 2019). Moreover, basic region/leucine zipper motif (bZIP) TFs regulate various biological processes, including signal transduction, defence responses, maturation and flower development (Jakoby et al. 2002). VIP1 is a classic bZIP protein regulating the mannitol responses (Tsugama et al. 2014). In the present study, a GY-highly expressed bHLH13, a PE-highly expressed WRKY33, and a GY-highly expressed VIP1, were identified in *C. goeringii*. Mining of floral organ differentially expressed TFs may help identify candidate regulators related to floral organ development.

## Conclusions

A comprehensive proteomic profile of *C. goeringii*, aimed at discovering proteins participating in floral organ patterning and development, has been developed. A total of 2,331 protein groups, of which 1,855 proteins were quantified, were identified in four floral organs of *C. goeringii*. A differential expression analysis showed that most DEPs were enriched in amino acid, lipid, and energy metabolism. Furthermore, hormone-related proteins were identified, suggesting a significant role of hormones in the regulation of flower morphogenesis and development. Three floral organ differentially expressed TFs, bHLH13, WRKY33 and VIP1, were identified, which will aid in the identification of candidate regulators related to floral organ development.

## Methods

### Plant material and sampling

Five-year-old *Cymbidium goeringii* seedlings were planted in a greenhouse at Zhejiang Academy of Agriculture Science at a temperature of 26 ± 1 °C with a light/dark cycle of 8/16 h and 65–75% relative humidity. In March 2019, samples from various floral organs, including the SE, PE, LI, and GY, were harvested for organ-specific proteomic analysis. Each sample was collected from 20 independent flowers and a pack of about 6–7 flowers was treated as one biological replicate. There were three biological replicates for each floral organ group.

### Protein isolation and extraction

An appropriate 500 mg of samples from each floral organ were ground into power in mortar with liquid N_2_. After adding of 500 µL lysis buffer, each sample was boiled for 5 min, sonicated for 5 min, and precipitated with trichloroacetic acid solution for 12 h. The lysis buffer contains 4% of sodium dodecyl sulfate, 100 mM Tris, 100 mM dithiothreitol and adjusted to pH 7.8. After 12,000×*g* centrifugation at 4 °C for 10 min, the supernatants were collected and the remaining debris was discarded. The protein samples were quantified by bicinchoninic acid assay (Walker 1994). Ten µg of each protein sample was added with loading buffer to 5:1 (v/v) and kept in boiling water bath for 5 min. Then, the resulting samples were checked by 8–16% SDS-polyacrylamide gel electrophoresis.

### Protein digestion and pretreatment

For protein digestion, 300 µg of each sample was added with dithiothreitol resulting in a final concentration of 100 mM. The samples were kept in boiling water bath for 5 min and cooled until room temperature. The samples were added with 200 µL of urea buffer containing 8 M urea and 150 mM Tris-HCl and centrifuged at 12,000×*g* for 10 min. After centrifugation, supernatant was discarded. Subsequently, the sample were shaken and alkylated with 100 µL of 50 mM iodoacetamide buffer for 1 min. After 30 min incubation in darkness, the precipitants were extracted by centrifugation at 12,000×*g* for 15 min. Then, samples were washed by 100 µL of urea buffer twice and followed by 100 µL of NH_4_HCO_3_ buffer twice. Finally, the resulting samples were added with 40 µL of trypsin buffer, containing 6 µg trypsin in 40 µL of NH_4_HCO_3_ buffer, shaken at 600 rpm at 37 ℃ for 16 h.

For desalination, the resulting samples were put into a tube by centrifugation at 12,000×*g* for 15 min, and added with 0.1% trifluoroacetic acid solution. The remaining samples were desalinated in a C18 Cartridge (Sigma-Aldrich).

### Tandem Mass Tag (TMT) labeling and fractionation of peptides

After peptide quantification, about 100 µg of each sample was harvested and labeled with one unit of TMT reagents as the provider’s instructions (Thermo Fisher Scientific). Briefly, the peptide samples were dissolved in 100 µL of 0.05 M tetraethylammonium bromide solution and the TMT reagents were dissolved in 41 µL of anhydrous acetonitrile solution, respectively. Then, the above two solutions were mixed and incubated for 1 h. The reaction was stopped by adding 8 µL of 5% hydroxylamine for 15 min.

The TMT-labeled peptide solution was separated by a Pierce™ High pH Reversed Phase Peptide Fractionation column on an Agilent high-performance liquid chromatographer (HPLC) system. Thirty fractions were harvested for each sample and concatenated to 15 fractions for further analysis (Yu et al. 2020).

### Liquid chromatography-tandem mass spectrometry (LC-MS/MS) analysis

After re-dissolution, protein samples were used for LC-MS/MS analysis. Samples were chromatographically separated by a nano flow rate EasynLC1200 chromatographic system (Thermo-Scientific). The solvent system consisting of Solution A (0.1% aqueous solution of formic acid) and solution B (0.1% formic acid and 85% acetonitrile). Chromatographic column was balanced with 95% of solution A at a flow rate of 0.75. The sample was uploaded to Trap C18 column (100 μm × 20 mm, 5 μm) and was separated by C18 chromatographic column (75 μm × 150 mm, 3 μm). The liquid phase separation gradient is set as follows: 0–5 min, B solution from 5 to 8%; 5–50 min, B solution from 8 to 23%; 50–60 min, B solution from 23 to 40%; 60–65 min, B solution from 40 to 100%; 65–75 min, B solution maintain at 100%. The following ion source parameters, including spray voltage 1.8 kV, capillary temperature 275 °C and declustering potential 100 V, were set. The mass spectrometer was run using a data-dependent Top-20 acquisition mode, switching automatically between MS and MS/MS.

After separation, data dependent acquisition MS was carried out by Q Exactive Plus mass spectrometer (Thermo Scientific). The essential parameters were shown as follows: analysis time is set at 75 min; detection mode is set at positive ion; parent ion scanning range is set from 300 to 1800 *m/z*; first stage mass spectrometry resolution (70,000) is set at *m/z* 200; AGC target is set at 3E6, and Level 2 maximum IT is set at 30 ms. Peptide secondary mass spectrometry analysis was performed according to the following sets: 20 highest intensity parent ions were collected after each full scan, second stage mass spectrometry resolution (17,500) is set at *m/z* 200, AGC target is set at 1e5, Level 2 MaTimum is set at 60 ms, and MS2 Activation Type is set at HCD.

### Database search

The raw LC-MS/MS data is uploaded into MaxQuant software (ver. 1.6.0.16) for database searching. The database used in the library is UniProt-Apostasia shenzhenica-21681-20181217, from the protein database on the web site https://www.uniprot.org/uniprot/?query=taTonomy:10090. The number of proteins is 21,681. A precursor mass 6 ppm was set as initial search. The main parameters are set as follows: isobaric label is TMT 6pleT, enzyme is Trypsin, reporter mass tolerance is set at 0.005 Da, MaT missed cleavage is 2, peptide search tolerance is set at 4.5 ppm, peptide tolerance of first round search is 20 ppm. MS/MS tolerance is 20 ppm, FiTed modification is carbamidomethyl, and variable modifications are OTidation and Acetyl. The mass spectrometry proteomics data have been deposited to the ProteomeXchange Consortium via the PRIDE partner repository with the dataset identifier PXD019636.

### Protein quantification and differentially expressed protein (DEPs)

The intensity ratios of the TMT reporter ions in MS/MS from raw data were applied to analyze the fold changes of each protein between two different groups. The expression level of each protein was mean-normalized to center the distribution of quantitative values. Protein quantitation was calculated as the median ratio of corresponding unique peptides. DEPs were screened in six different comparisons with cut-off of ratio fold as > 1.2 or < 0.83 and *P* values < 0.05. Expression levels of proteins were analyzed by hierarchical clustering method. To reach the criterion of the hierarchical clustering, the significant *P* values were transformed into *Z*-score by log transformation.

### Enrichment analysis of the DEPs

A two-tailed Fisher’s exact text was performed to analyze Gene Ontology (GO) and Encyclopedia of Genes and Genomes (KEGG) enrichment of the DEPs against all identified proteins. The categories that were at least enriched in one of the clusters with *P* value < 0.05 were filtered out, and the filtered *P* values were transformed by the formula: adjusted *P* = −log10 (*P* value).

## Supplementary Information

**Additional file 1: Table S1.** The basic information of all identified proteins.

**Additional file 2: Table S2. **The basic information of amino acid metabolism-related proteins.

**Additional file 3: Table S3. **The basic information of fatty acid metablism-related proteins.

**Additional file 4: Table S4. **The basic information of energy metabolism-related proteins.

**Additional file 5: Table S5.** The basic information of cell division and pigment related proteins.

## Data Availability

The mass spectrometry proteomics data have been deposited to the ProteomeXchange Consortium via the PRIDE partner repository with the dataset identifier PXD019636.
